# Work loss among privately insured employees with overweight and obesity in the United States

**DOI:** 10.1002/osp4.775

**Published:** 2024-07-08

**Authors:** Shraddha Shinde, Anh Thu Tran, Michelle Jerry, Clare J. Lee

**Affiliations:** ^1^ Eli Lilly and Company Indianapolis Indiana USA; ^2^ Merative Ann Arbor Michigan USA

**Keywords:** absenteeism, overweight and obesity, short‐ and long‐term disability, workers'compensation

## Abstract

**Background:**

Rising obesity rates in the workforce are accompanied by a hidden cost burden to employers due to work productivity loss. Understanding the impact of obesity on work productivity is essential for employers to provide tailored weight loss interventions in the workplace.

**Objectives:**

To measure work loss and associated productivity costs among employees with overweight/obesity compared with employees with normal weight.

**Methods:**

This retrospective cohort study used the Merative^TM^ MarketScan® Health and Productivity Management Database to identify adult employees with ≥1 diagnosis code reporting a body mass index (BMI) between 1/1/2015‐12/31/2019. Based on the earliest BMI, employees were assigned to normal weight (BMI 19–24.9), overweight (BMI 25–29.9), obesity class 1 (BMI 30–34.9), obesity class 2 (BMI 35–39.9), and obesity class 3 (BMI ≥40) cohorts. Among employees with data for each work loss category (absenteeism, short‐term disability [STD], long‐term disability [LTD], worker's compensation [WC]) during the 12‐month follow‐up, the percentage of employees with work loss, number of hours/days lost, and associated productivity costs were reported.

**Results:**

719,482 employees (normal weight: 106,631, overweight: 230,637, obesity class 1: 185,850, obesity class 2: 101,909, obesity class 3: 94,455) were included. Outcomes increased with each higher BMI category for the mean number of absence hours ([in order of BMI category]: 262, 273, 285, 290, 304) and percentage of employees with a claim (STD: 6.8%, 7.6%, 9.7%, 11.7%, 17.0%; LTD: 0.4%, 0.4%, 0.5%, 0.6%, 0.8%; WC: 2.7%, 2.8%, 3.4%, 3.6%, 3.5%). Estimated costs to the employer associated with absenteeism, STD, LTD, and WC were $1,036, $611, $38, and $95 higher per year (respectively) in the obesity class 3 cohort relative to the normal weight cohort.

**Conclusions:**

This real‐world analysis demonstrated that employees with overweight/obesity had higher loss of work productivity compared with employees with normal weight. Further studies are warranted to determine the long‐term impacts on work productivity loss if overweight and obesity are left untreated.

## 1 BACKGROUND

Obesity is a significant public health issue with a steadily rising prevalence from 31% of the United States (US) adult population in 1999‐2000 to 42% of the US adult population in 2017–2020.^1^ Obesity is associated with numerous conditions, including heart disease, stroke, and type 2 diabetes, which are among the leading causes of preventable morbidity and mortality.^2^ A systematic review of 50 studies published between January 2000 and June 2017 concluded that there are substantial short‐term and long‐term indirect costs associated with overweight and obesity.^3^ In 2016, the US total costs of chronic diseases related to overweight and obesity amounted to $1.72 trillion, comprising $480.7 billion in direct healthcare costs and $1.24 trillion in indirect costs attributable of work productivity impairment.^4^ Yet real‐world data are limited on the impacts of overweight and obesity on work productivity of employees and associated indirect costs incurred to employers in the US.

Several studies have estimated the costs of workplace absenteeism associated with overweight and obesity using data from the US National Health and Wellness Survey, a self‐administered questionnaire including absence hours at work.^5^, ^6^, ^7^ Based on the survey data, Finkelstein et al. estimated annual excess absenteeism costs associated with overweight or obesity in 2008 to range from $85 to $1262 per employee, compared to employees with normal weight; additionally, the annual absenteeism cost attributable to obesity among full‐time employees was $12.8 billion.^6^ Kudel et al. also noted the positive association between increases in body mass index (BMI) category and work productivity impairment across various occupations using the survey data from 2014 to 2015, with the greatest impacts on work productivity occurring among employees in the construction, arts and hospitality industries.^7^

Studies leveraging claims and employer databases have also estimated the costs of work productivity loss associated with overweight and obesity.^8^, ^9^ A study by Kleinman et al. quantified the impact of overweight and obesity on attributable indirect costs related to absenteeism (due to sick leave), short‐term disability (STD), long‐term disability (LTD), and workers’ compensation (WC) using 2003 to 2011 claims data from a large US employer database (Human Capital Management Services Research Reference Database). The annual average total productivity costs (for absenteeism, STD, LTD, and WC combined) were estimated to be $379 higher among employees with obesity (BMI ≥30 kg/m^2^) relative to employees with BMI <27 kg/m^2^ ($1332 vs. $953).^8^ More recently, Ramasamy et al. used data from the Optum Health Reporting and Insights employer claims database from 2010 to 2017 to evaluate the obesity‐related absenteeism, STD, and LTD costs among privately insured employees, finding that annual productivity costs (for medical‐related absenteeism, STD, and LTD combined) were $617, $541, and $1707 higher for employees with obesity classes 1, 2, and 3, respectively, relative to employees with normal weight.^9^

Overall, previous studies have examined the association between work productivity loss and overweight and obesity.^5^, ^6^, ^7^, ^8^, ^9^ However, these studies used less recent real‐world data (up to 2017)^5^, ^6^, ^7^, ^8^, ^9^ and only one study evaluated all categories of work loss, including absenteeism, STD, LTD, and WC.^8^ To provide a more comprehensive and updated understanding of the impact of overweight and obesity on work productivity loss and indirect costs, especially amidst rapidly rising rates of obesity in the US, this retrospective cohort study used administrative claims and employer data from 2015 to 2019 to examine work loss and associated productivity costs (including absenteeism, STD, LTD, and WC) among employees with normal weight, overweight, and obesity (classes 1, 2, and 3) in the US.

## 2 MATERIALS AND METHODS

### 2.1 Study design and data source

This retrospective, observational cohort study utilized work productivity data (including absenteeism, STD, LTD, and WC) from the Merative^TM^ MarketScan® Health and Productivity Management (HPM) Database. This database contains workplace absence, STD, LTD, and WC data, which can be linked to medical and pharmacy utilization data from large US employers. Not all employers that contribute to this database report all types of productivity data. Presenteeism data and specific reasons for employee's retirement are not available in the database. Data were obtained using the International Classification of Diseases, ninth or 10th Revision, Clinical Modification (ICD‐9‐CM or ICD‐10‐CM) codes, Procedure Coding System (ICD‐9‐PCS or ICD‐10‐PCS) codes, Current Procedural Terminology codes, Healthcare Common Procedure Coding System codes, and National Drug Codes. Because the study used only de‐identified patient records (and did not involve the collection, use, or transmittal of individually identifiable data), Institutional Review Board approval to conduct this study was not necessary.

### 2.2 Sample selection

Eligible employees were required to have ≥1 medical claim with a diagnosis code for a BMI ≥19.0 (ICD‐9‐CM codes: V85.0 to V85.4X; ICD‐10‐CM codes: Z68.2 to Z68.45) between 1/1/2015 and 12/31/2019 (earliest claim date = index date). Additional inclusion criteria included: (1) ≥18 years of age on the index date, (2) continuous enrollment with medical and pharmacy benefits for 12‐month pre‐index (baseline) and 12‐month post‐index (follow‐up) periods, (3) full‐time or part‐time employment status as of the index date (to ensure that study outcomes were only collected for active employees and not individuals on LTD or temporary employer‐sponsored health coverage after job loss), and (4) available data in the HPM database for at least one category of work loss. Employees were excluded if they had claims with different BMI values on the index date or claims for pregnancy during the full study period 1/1/2014–12/31/2020 (including the baseline and follow‐up periods).

Included employees were classified into cohorts based on the BMI value on the index claim with BMI thresholds defined by the Centers for Disease Control and Prevention (CDC): normal weight cohort (BMI 19.0–24.9 kg/m^2^), overweight cohort (BMI 25.0–29.9 kg/m^2^), obesity class 1 cohort (BMI 30.0–34.9 kg/m^2^), obesity class 2 cohort (BMI 35.0–39.9 kg/m^2^), and obesity class 3 cohort (BMI ≥40 kg/m^2^). Although BMI values for normal weight are in the range of 18.5–24.9 per the CDC, the BMI range for the normal weight cohort was modified to 19.0–24.9 because ICD‐9‐CM and ICD‐10‐CM diagnosis codes are not available at the required level of granularity for BMI <19.0.

### 2.3 Outcomes

The primary outcomes were work loss and associated productivity costs measured during the 12‐month follow‐up period. For each work loss category (absenteeism, STD, LTD, or WC), the proportion of employees with ≥1 record of work loss, number of hours/days lost (assuming an 8‐h workday and a 5‐day workweek), and attributable productivity costs associated with work loss were reported among employees with data available for the respective work loss category. Because employers that contribute to the HPM database may contribute only some types of absence (e.g., sick, recreational, etc.), having zero absence hours may be influenced by the types of absenteeism data contributed by the given employee's employer. Thus, the analysis for absenteeism was restricted to employees with ≥1 absence hour to limit this potential bias. Based on the number of hours/day lost documented on claims, attributable productivity costs were estimated by multiplying hours/days lost by a percentage of the 2020 median daily wage ($196.80/day) for full‐time wage and salary workers reported by the US Bureau of Labor Statistics.^10^ In general, wages are paid 100% for workplace absence, 70% for STD, 60% for LTD, and 67% for WC,^11^ so these percentages were used to compute costs.

Demographics were reported on the index date, including age, sex, geographic region, insurance plan type, index year, employer industry, employee classification (salary vs. hourly), and employment status (full‐time vs. part‐time). Baseline clinical characteristics were measured during the 12‐month baseline period, including Charlson Comorbidity Index (CCI), weight‐related comorbidities (type 2 diabetes, hyperlipidemia, hypertension, cardiovascular disease, and sleep apnea), other comorbidities (low back pain, osteoarthritis, anxiety, depression, asthma, atrial fibrillation, chronic kidney disease, heart failure, infertility, knee replacement surgery, myocardial infarction, non‐alcoholic fatty liver disease, non‐alcoholic steatohepatitis, gout, obesity‐related cancer, polycystic ovarian syndrome, unstable angina, and urinary incontinence), and number of these comorbidities (0, 1, 2+ comorbidities). Obesity‐related treatments were captured during the 12‐month follow‐up, including evidence of bariatric surgery (gastric bypass, gastric banding, sleeve gastrectomy, and other procedures including implantation and/or revision for gastric balloon, gastric neuromodulator, or vagus nerve stimulator) and use of anti‐obesity medications (topiramate, metformin [among individuals without a diagnosis for type 2 diabetes during the follow‐up], phentermine, liraglutide, naltrexone‐bupropion, lorcaserin, phentermine‐topiramate, phendimetrazine, diethylpropion, orlistat, and benzphetamine). Clinical measures were documented based on the evidence of at least one medical claim with a diagnosis or procedure code for the comorbidity or bariatric surgery of interest, respectively, and at least one pharmacy claim with a drug code for anti‐obesity medications. Additionally, CCI and number of comorbidities were stratified by sex and age category (among all included patients and each weight cohort) to investigate whether observed differences in work loss by sex and age category may be related to differences in comorbidity burden within these sex and age subgroups.

### 2.4 Statistical analysis

Descriptive analysis was conducted to report the demographics, clinical characteristics, and productivity loss outcomes of each weight cohort. Categorical variables were presented as the count and percentage of individuals in each category. Continuous variables were summarized by providing mean, standard deviation, and median. The normal weight cohort was compared with each of the other cohorts using Chi‐square tests for categorical variables and Student's *t*‐tests for continuous variables. A critical value of 0.05 was specified a priori as the threshold for statistical significance.

Multivariable two‐part models were constructed to examine the association between the BMI category and productivity loss outcomes after adjusting for differences in demographics. The first part (logistic regression) assessed the probability of having a work loss claim. The second part (generalized linear model with log link and gamma error distribution) assessed the number of hours/days lost among employees with a work loss claim. For the absenteeism analysis, only the second part was needed (since employees were required to have ≥1 absence hour). For all models, covariates included BMI category, age, sex, employer industry, and employment status. Based on the output of multivariable two‐part models, recycled predictions^12^ were used to calculate the number of hours/days lost and attributable productivity costs for each weight cohort after adjusting for baseline differences. Comorbidities and CCI score were not included in the models for two reasons. First, the list of comorbidities reported in this study is not comprehensive; therefore, adjusting for these comorbidities would only adjust for a subset of comorbidities that may be related to work loss. Second, and more importantly, employees with overweight and obesity will develop comorbidities as a result of their weight and will take more time off from work due to those comorbidities, so adjusting for comorbidities or CCI score would adjust away the treatment effect that the study tried to observe.

## 3 RESULTS

### 3.1 Study population

**FIGURE 1:**
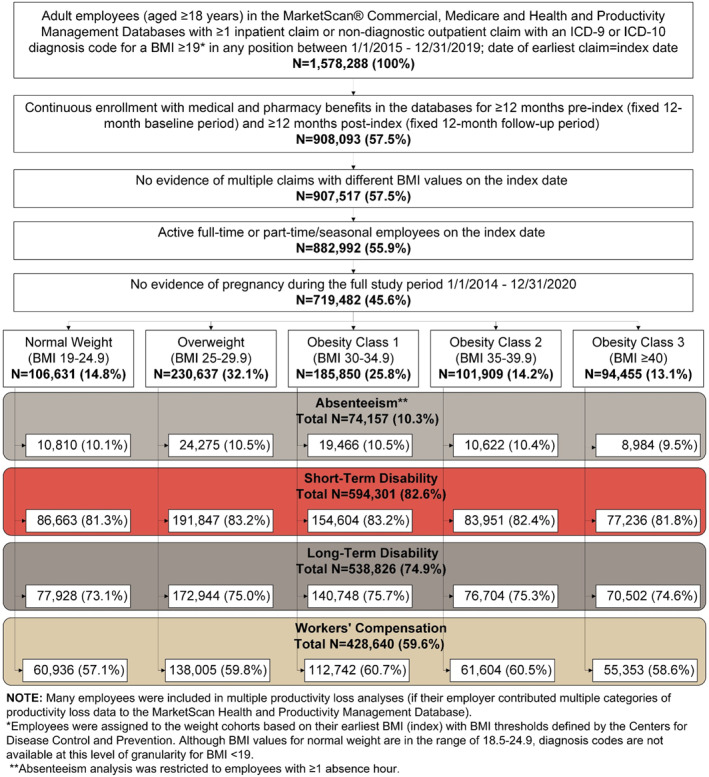
Sample attrition flowchart costs.

A total of 719,482 employees (14.8% normal weight, 32.1% overweight, 25.8% obesity class 1, 14.2% obesity class 2, 13.1% obesity class 3) were included in at least one of the analyses related to productivity loss. Approximately 10%, 83%, 75%, and 60% of all included employees had data available for analysis of absenteeism, STD, LTD, and WC, respectively (Figure 1). The mean age ranged from 45.4 to 48.0 years and more than half of employees were male (range: 55.0%–72.0%; Table 1). Employees in the overweight, obesity class 1, and obesity class 2 cohorts were older and more likely to be male compared to employees with normal weight (*p* < 0.001). The most common industry was manufacturing/durable goods (range: 26.0%–31.6%) and almost all employees worked full‐time (≥97.2%).

**TABLE 1:** Patient characteristics.

	Normal weight	Overweight	Obesity class 1	Obesity class 2	Obesity class 3
	*N* = 106,631	*N* = 230,637	*N* = 185,850	*N* = 101,909	*N* = 94,455
**Demographics**					
Age (mean ± SD)	45.4 ± 11.2	47.2 ± 10.3	48.0 ± 9.8	47.7 ± 9.8	46.7 ± 9.8
Age category^a^ (*N*, %)					
18–34	21.4%	14.9%	11.9%	12.2%	13.8%
35–44	22.0%	22.1%	21.8%	22.5%	25.1%
45–54	30.1%	33.6%	35.4%	36.2%	35.4%
55–65	26.2%	29.1%	30.5%	28.8%	25.4%
65–74	0.3%	0.4%	0.4%	0.3%	0.2%
Male (%)	55.1%	72.0%	70.1%	63.2%	55.0%
Geographic region (*N*, %)					
Northeast	16.3%	15.8%	14.6%	14.1%	13.1%
North central	27.9%	27.8%	28.2%	28.4%	29.3%
South	42.5%	44.8%	45.6%	45.9%	44.4%
West	13.1%	11.4%	11.4%	11.4%	13.1%
Unknown	0.2%	0.2%	0.2%	0.2%	0.1%
Insurance plan type (*N*, %)					
Comprehensive/indemnity	4.8%	4.6%	4.6%	4.6%	4.0%
EPO/PPO	45.3%	45.6%	46.9%	48.2%	48.5%
POS/POS with capitation	2.4%	2.4%	2.9%	3.3%	3.8%
HMO	10.2%	10.6%	11.4%	11.6%	11.9%
CDHP/HDHP	36.6%	36.0%	33.4%	31.7%	31.1%
Other/unknown	0.7%	0.7%	0.7%	0.7%	0.7%
Index year (*N*, %)					
2015	25.2%	24.2%	23.7%	22.6%	24.5%
2016	19.9%	21.1%	21.5%	21.8%	22.8%
2017	20.0%	20.4%	20.0%	20.1%	19.3%
2018	18.5%	18.3%	18.6%	19.5%	18.0%
2019	16.3%	15.9%	16.2%	16.0%	15.3%
Employer industry (%)					
Manufacturing, durable goods	29.1%	31.6%	31.5%	29.7%	26.0%
Manufacturing, nondurable goods	9.2%	9.5%	9.5%	8.9%	7.6%
Transportation, communications, utilities	15.7%	19.3%	20.9%	21.1%	20.7%
Finance, insurance, real estate	18.6%	16.4%	14.3%	14.0%	16.1%
Services	18.1%	14.8%	14.9%	16.8%	19.3%
Retail trade	6.2%	4.9%	5.0%	5.3%	5.7%
Oil & gas extraction, mining	1.1%	1.3%	1.2%	1.1%	1.0%
Wholesale	0.4%	0.4%	0.5%	0.5%	0.6%
Construction	0.1%	0.2%	0.2%	0.2%	0.1%
Agriculture, forestry, fishing	0.1%	0.1%	0.1%	0.1%	0.1%
Unknown	1.4%	1.6%	2.0%	2.3%	2.8%
Employee classification (%)					
Salary	50.5%	47.6%	41.7%	38.3%	35.8%
Hourly	36.2%	38.2%	43.2%	45.6%	47.0%
Unknown	13.3%	14.2%	15.2%	16.1%	17.2%
Employee status (%)					
Active full‐time	97.2%	98.2%	98.2%	98.1%	97.9%
Active part‐time/seasonal	2.8%	1.8%	1.8%	1.9%	2.1%
**Baseline clinical characteristics (during 12‐month baseline)**					
CCI (Mean ± SD)	0.3 ± 0.9	0.4 ± 1.0	0.5 ± 1.1	0.6 ± 1.2	0.7 ± 1.3
Weight‐related comorbidities (%)					
Hypertension	11.4%	20.6%	31.5%	39.1%	46.1%
Hyperlipidemia	13.7%	22.1%	27.9%	29.9%	28.8%
Type 2 diabetes	2.9%	5.6%	10.6%	15.4%	19.8%
Sleep apnea	1.4%	3.9%	8.8%	14.9%	23.4%
Cardiovascular disease	2.6%	3.5%	4.4%	4.6%	4.6%
Select other comorbidities^b^ (%)					
Low back pain	11.0%	12.3%	13.7%	14.5%	15.9%
Anxiety	8.9%	8.5%	8.7%	9.2%	9.5%
Osteoarthritis	3.6%	5.0%	7.1%	9.2%	12.6%
Depression	5.5%	5.7%^c^	6.5%	8.0%	9.7%
Asthma	3.4%	3.8%	4.7%	6.0%	7.8%
Number of comorbidities^d^ (%)					
0 comorbidity	59.0%	48.9%	38.5%	31.8%	25.8%
1 comorbidity	23.2%	24.2%	23.8%	22.8%	21.3%
2+ comorbidities	17.8%	26.9%	37.6%	45.4%	53.0%
Obesity‐related treatments (during 12‐month follow‐up)					
Anti‐obesity medications^e^ (%)					
Any anti‐obesity medication	1.23%	1.84%	3.78%	5.78%	8.05%
Topiramate	0.86%	0.88%^b^	1.31%	1.82%	2.49%
Metformin^f^	0.28%	0.59%	1.23%	1.92%	2.92%
Phentermine	0.06%	0.22%	0.62%	0.98%	1.18%
Liraglutide	0.03%	0.11%	0.37%	0.66%	1.00%
Naltrexone‐bupropion	0.01%	0.07%	0.30%	0.52%	0.75%
Lorcaserin	0.01%	0.05%	0.18%	0.30%	0.43%
Bariatric surgeries^g^ (%)					
Any bariatric surgery	0.01%	0.02%^b^	0.11%	1.44%	6.16%
Sleeve gastrectomy	0.00%	0.01%^b^	0.07%	1.31%	5.81%
Gastric banding	0.00%	0.00%^b^	0.00%^b^	0.05%	0.17%
Other	0.01%	0.01%^b^	0.05%	0.15%	0.39%

Abbreviations: CCI, Charlson comorbidity index; CDHP, consumer‐driven health plan; EPO, exclusive provider organization; HDHP, high deductible health plan; HMO, health maintenance organization; POS, point of service; PPO, preferred provider organization; SD, standard deviation.

^a^ None of the included employees were older than 74 years.

^b^ Additional comorbidities included atrial fibrillation, chronic kidney disease, heart failure, infertility, knee replacement surgery, myocardial infarction, non‐alcoholic fatty liver disease, non‐alcoholic steatohepatitis, gout, obesity‐related cancer, polycystic ovarian syndrome, unstable angina, and urinary incontinence. Each of these comorbidities was present in less than 1.5% of all included employees.

^c^ *p*‐values were not significant (all *p* > 0.05) in comparison to the normal weight cohort.

^d^ Included the weight‐related comorbidities, other documented comorbidities.

^e^ Included the anti‐obesity medications reported in Table 1 plus the following: phentermine‐topiramate, phendimetrazine, diethylpropion, orlistat, and benzphetamine. Semaglutide indicated for obesity and setmelanotide were approved at the end of the study period and were not considered in this analysis.

^f^ Metformin was documented among employees without a diagnosis of type 2 diabetes during the 12‐month follow‐up.

^g^ Gastric bypass was not observed in any of the included employees. Other bariatric procedures included implantation and/or revision of gastric balloons, gastric neuromodulators, or vagus nerve stimulators.

*p*‐values of normal weight cohort versus each other weight cohort were significant (all *p* < 0.001) except where noted.

A low comorbidity profile was observed with mean CCI scores ranging from 0.3–0.7 at baseline, and the CCI score increased with each higher BMI category (all *p* < 0.001; Table 1). The prevalence of weight‐related comorbidities also increased with each higher BMI category (all *p* < 0.001; except for hyperlipidemia which increased to a peak for obesity class 2 before declining slightly for the obesity class 3 cohort). Hypertension (range: 11.4%–46.1%) and hyperlipidemia (range: 13.7%–29.9%) were the most common weight‐related comorbidities. During the 12‐month follow‐up, few employees with overweight and obesity used anti‐obesity medications (range: 1.84%–8.05%) or received bariatric surgeries (range: 0.02%–6.16%) (Table 1). Rates of medication utilization and surgeries generally increased with each higher BMI category, with topiramate, metformin, and sleeve gastrectomy being the most common medications and surgeries, respectively.

### 3.2 Absenteeism

**FIGURE 2:**
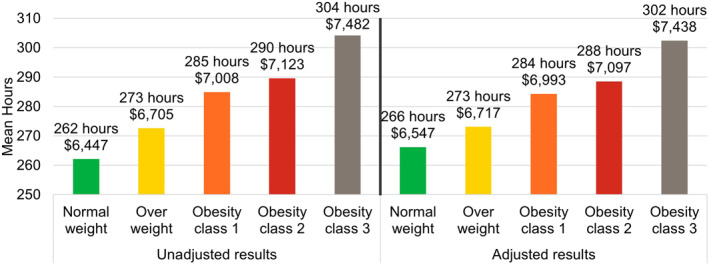
Absenteeism outcomes: Annual number of hours lost and attributable productivity costs. Costs were estimated by multiplying the number of days lost by a percentage of wages. Covariates included in the adjustment were age, sex, employer industry, and employment status (part‐time vs. full‐time).

The analysis of absenteeism included 74,157 employees (10.3% of overall population; Figure 1). The mean number of absence hours and attributable productivity costs increased with each higher BMI category both before and after adjustment (Figure 2). Employees with obesity class 3 had higher mean annual hours of absence and costs compared to employees with normal weight (unadjusted difference: 42 h, $1036; adjusted difference: 36 h, $891; all *p* < 0.001).

After adjustment and compared with the normal weight cohort, employees in the overweight, obesity class 1, obesity class 2, and obesity class 3 cohorts had 3%, 7%, 8%, and 14% higher hours of workplace absence, respectively (all *p* < 0.001; Table S1). Multivariable modeling also showed that sex was not significantly associated with higher absence hours, but each decade of higher age was associated with 8% higher hours of absence (*p* < 0.001). For the employer industry, compared to manufacturing of durable goods, manufacturing of nondurable goods, transportation/communications/utilities, and oil & gas extraction/mining were associated with 32%, 19%, and 19% lower hours of workplace absence, respectively (all *p* < 0.001).

### 3.3 Short‐term disability, long‐term disability, and workers' compensation

**FIGURE 3:**
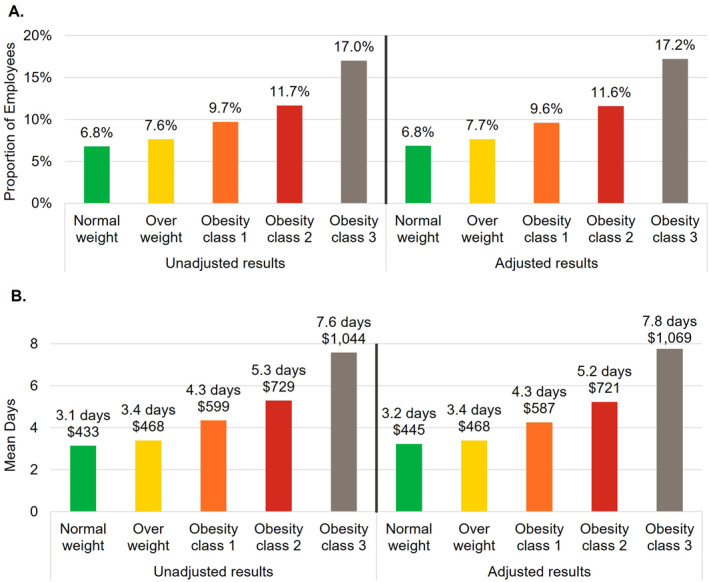
Short‐term disability (STD) outcomes. (A) Annual proportion of employees with a STD claim. (B) Annual number of days lost and attributable productivity costs associated with short‐term disability. Costs were estimated by multiplying the number of days lost by a percentage of wage. Covariates included in the adjustment were age, sex, employer industry, and employment status (part‐time vs. full‐time).

**FIGURE 4:**
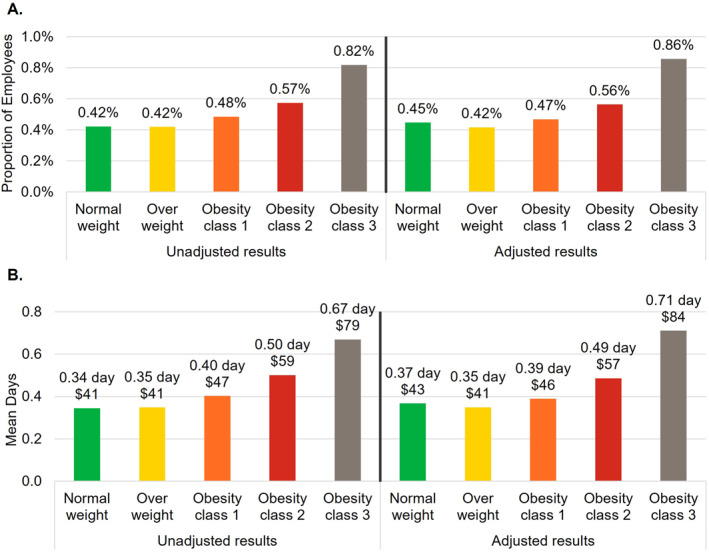
Long‐term disability (LTD) outcomes. (A) Annual proportion of employees with a LTD claim. (B) Annual number of days lost and attributable productivity costs associated with LTD. Costs were estimated by multiplying the number of days lost by a percentage of wage. Covariates included in the adjustment were age, sex, employer industry, and employment status (part‐time vs. full‐time).

**FIGURE 5:**
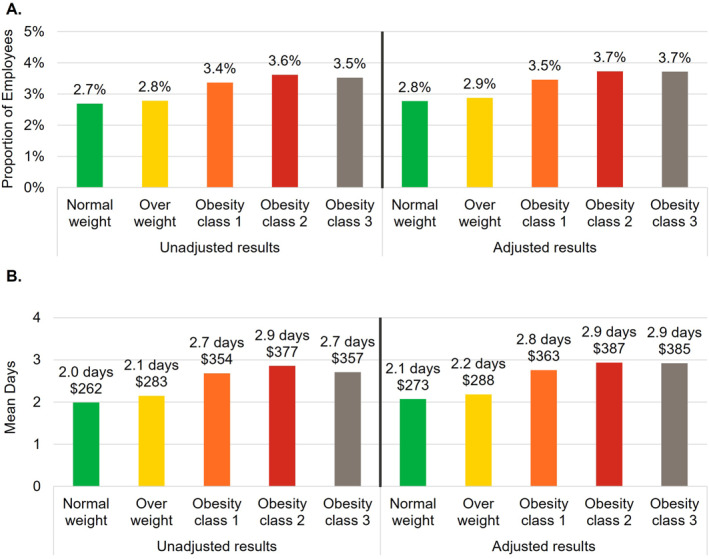
Workers' compensation outcomes. (A) Annual proportion of employees with a workers' compensation claim. (B) Annual number of days lost and attributable productivity costs associated with workers' compensation. Costs were estimated by multiplying the number of days lost by a percentage of wage. Covariates included in the adjustment were age, sex, employer industry, and employment status (part‐time vs. full‐time).

The number of employees included in the remaining work loss analyses were 594,301 (82.6% of overall population) for STD, 538,826 (74.9% of overall population) for LTD, and 428,640 (59.6% of overall population) for WC (Figure 1). For each type of work loss, outcomes generally increased with each higher BMI category both before and after adjustment (Figures 3, 4, 5). Employees in obesity class 3 had higher proportions with a STD, LTD, and WC claim, respectively, compared to employees with normal weight (unadjusted difference: STD 10.2%, LTD 0.4%, and WC 0.8%; adjusted difference: STD 10.4%, LTD 0.4%, and WC 0.9%; all *p* < 0.001). The mean annual number of days lost and costs for STD, LTD, and WC were higher among employees with obesity class 3 compared to employees with normal weight and were more than double for STD and nearly double for LTD (unadjusted differences: STD 4.4 days, $611; LTD 0.32 days, $38; WC 0.7 days, $95; adjusted differences: STD 4.5 days, $623; LTD 0.34 days, $41; WC 0.9 days, $112; all *p* < 0.001). Because only 0.4%–0.8% of employees (depending on BMI category) had an LTD claim, costs associated with LTD per employee were low in relation to costs for absenteeism, STD, and WC.

After adjustment and compared with the normal weight cohort, employees with obesity class 3 had 188%, 93%, and 36% higher odds of having a STD, LTD, and WC claim, respectively (all *p* < 0.001). Moreover, multivariable models also showed significantly higher odds of having a work loss claim for each decade higher age (STD 16%, LTD 50%, WC 4%; all *p* < 0.001) and female gender (STD 37%, LTD 25%, WC 18%; all *p* < 0.001; Table S2). For the employer industry, compared to manufacturing of durable goods, other industries (except transportation/communication/utilities) were associated with significantly lower odds of having a STD, LTD, and WC claim. Retail trade had the lowest odds of having a STD and LTD claim (72% and 86% lower odds of having a STD or LTD claim relative to the employees in the manufacturing of durable goods industry, respectively), and no retail trade employees had a WC claim.

Post hoc reporting of comorbidity burden among female versus male employees and by age category revealed that comorbidity burden is higher among females and higher with each increasing age category (both overall and within each BMI category) (Figures S1 and S2). This higher comorbidity burden may explain the reason why higher age and female gender were significantly associated with higher work loss.

## 4 DISCUSSION

This retrospective cohort study added more recent data (up to 2019) and more comprehensive insights on the employers' economic burden of overweight and obesity in their employees and associated productivity loss in the US. To the authors' knowledge, there is no recent study that evaluates the employee productivity loss related to each of absenteeism, STD, LTD, and WC (prior study included data up to 2011^8^). Additionally, outcomes were evaluated among a much larger sample of US employees than what was previously available in the literature. Previous studies either used survey data or employee data and mainly focused on absenteeism, STD, and LTD costs.^5^, ^6^, ^7^, ^8^, ^9^ In general, this study found that each higher BMI category was associated with an increase in work loss.

Similar trends of higher costs associated with overweight and obesity were observed in the previous studies.^8^, ^9^ Using data from the Optum Health Reporting and Insights employer claims database from 2010 to 2017, Ramasamy et al. reported that annual productivity costs (for medical‐related absenteeism, STD, and LTD combined) were $617, $541, and $1707 higher for employees with obesity classes 1, 2, and 3, respectively, relative to employees with normal weight.^9^ These findings are consistent with the current study where employees with obesity classes 1, 2, and 3 had $445, $550, $891 higher absenteeism costs; $141, $276, $623 higher STD costs; and $3, $14, $41 higher LTD costs, respectively, relative to employees with normal weight, despite using different employee databases (with inherently different compositions of employee industry and type) and different methodologies for sample selection, cost components, and cost calculations. Ramasamy et al. captured medical‐related absenteeism (imputed based on healthcare utilization), while this study captured absenteeism reported by the employer that may be due to various reasons (including sick leaves, vacation, etc.). Additionally, productivity costs were calculated using the employees' recorded wages in the study of Ramasamy et al., while costs were estimated by multiplying hours/days of work loss by a median wage constant in this study.

For WC, a study by Kleinman et al. was the only study to the authors' knowledge that had reported the costs attributable to WC among employees with obesity from a large US employer database (Human Capital Management Services Research Reference Database). Using 2003 to 2011 claims data, Kleinman et al. found that employees with obesity (BMI ≥30 kg/m^2^) had an annual average of $59 higher WC costs relative to employees with BMI <27 kg/m^2^, which included medical payments and indemnity payments.^8^ While this study reported higher relative WC costs than those reported by Kleinman et al. ($91, $114, and $112 higher for employees with obesity classes 1, 2, and 3 relative to employees with normal weight), some of this difference may be attributed to inflation. Similarly, the annual productivity costs related to absenteeism, STD, and LTD in this study (listed in the previous paragraph) were considerably higher than those reported by Kleinman et al. ($242 for absenteeism, $71 for STD, and $7 for LTD), underscoring the need for the updated data provided in this study.

This study attempted to evaluate work productivity impairment by the employer industry. Compared with manufacturing of durable goods, manufacturing of nondurable goods was associated with the lowest hours of workplace absence and retail trade was associated with the lowest odds of having a STD, LTD, and WC claim (no employees in the retail trade industry had a WC claim). However, the absence hours by employer industry should be interpreted with caution because industry may not only reflect differences in absenteeism by industry but also serve as a surrogate for the employers that contributed their data to the MarketScan HPM Database. Some data contributors provide all types of absenteeism (e.g., sick, recreational, etc.) while others only report some types. Thus, lower absenteeism hours among the manufacturing of nondurable goods industry may be due to employers in this industry only contributing sick time to the MarketScan HPM Database (rather than both sick and recreational), for example. Regardless, it is difficult to determine why the burden of work productivity loss may be higher in one industry compared with another. Kudel et al. reported the highest absenteeism and presenteeism costs in employees working in construction.^7^ Ramasamy et al. observed the highest odds of incurring high medical‐related absenteeism and disability costs in employees of the government, education, and religious services.^9^ Taken together, findings from the previous and current studies suggest differing degrees of obesity‐related work productivity impairment across various industries, but additional research is needed to better understand the relationship between employer industry and obesity‐related work productivity loss.

In addition to the association between the BMI category and work loss in this study, female gender and older age were also associated with significantly higher odds of having a claim for STD, LTD, and WC, and older age was significantly associated with higher absence hours. The impacts of gender and age on work productivity have been reported in previous studies. Days lost and costs in all work loss categories were generally higher in females than males.^6^, ^8^ Also, in the Kleinman et al. study, when compared to younger employees, older employees had more days lost and greater costs related to STD, LTD, and WC but fewer days lost and lower costs for sick leave.^8^ The higher work loss associated with female gender and older age may be related to the higher comorbidity burden in these populations as evaluated in this study's post hoc analysis. These findings suggest that females, older individuals, and individuals with several comorbidities represent the population who may have the greatest opportunity for productivity improvement.

Although the substantial impacts of overweight and obesity have been consistently demonstrated in literature through not only the indirect costs for work loss but also the direct costs for healthcare expenditures, the management of obesity with pharmacotherapy and bariatric surgery was minimal in the current study, suggesting a potential gap in clinical care.^2^, ^5^, ^6^, ^8^, ^9^, ^13^, ^14^ The standard of care for overweight and obesity consists of lifestyle therapy (including healthy diet and physical activity), pharmacotherapy (recommended for individuals with BMI ≥30 kg/m^2^ or BMI ≥27 kg/m^2^ in presence of a weight‐related comorbidity) and bariatric surgery (reserved for individuals with BMI ≥40 kg/m^2^ or BMI ≥35 kg/m^2^ in presence of a severe weight‐related comorbidity).^15^ Lifestyle modifications remain the cornerstone of treatment for these conditions, while pharmacotherapy and bariatric surgery are adjunct therapies for achieving and sustaining weight loss in treatment‐approved populations.^15^ Research suggests that the use of anti‐obesity medications in conjunction with a weight management plan has resulted in a higher proportion of individuals achieving weight loss compared to those on a weight management plan alone.^16^, ^17^

Providing coverage for anti‐obesity medications, along with other advanced therapies and wellness programs for weight loss, is a method by which employers can support employees in combating obesity earlier in an effort to prevent weight‐related comorbidities from developing. Expanded health insurance coverage and increased uptake of anti‐obesity medications for appropriate patients may thereby lead not only to better health outcomes but also improved work productivity.^18^ Future research is needed to re‐evaluate the uptake of anti‐obesity medications in recent years (as additional anti‐obesity medications have become available) and the resulting long‐term impact on health and work productivity.

The limitations of this study included those inherent in any retrospective analysis. First, MarketScan HPM data were derived from a non‐random sample of large employers. Consequently, study findings of productivity loss outcomes may not be generalizable to the US employees working for small or mid‐sized employers. Second, the potential for misclassification of BMI category, covariates, or study outcomes was present as patients were identified through administrative claims data as opposed to medical records. As with any claims database, the MarketScan Research Databases rely on administrative claims data for clinical detail. These data are subject to data coding limitations and data entry errors. Third, this study included only employees whose BMI was captured in their administrative claims data. Individuals who have normal weight, are overweight, and have obesity whose BMI is not recorded in claims are not represented. In particular, individuals with normal weight may have underreported BMIs in claims because clinicians are less likely to document a normal BMI, contributing to the relatively small size of the normal weight cohort (14.8%) relative to the other cohorts. Fourth, multivariable analysis adjusted for differences in baseline characteristics across weight cohorts that were measurable in the databases and therefore was unable to account for other factors that may have influenced time away from work, such as employer‐specific allowances for time off. Fifth, an employee's comorbidities and CCI intentionally were not included as adjustment variables in the multivariable analysis because inclusion of these variables would effectively offset the expected additional work productivity burden that employees with overweight and obesity have as a result of their additional comorbidities; future research is needed to quantify the relationship between increased numbers of comorbidities and work productivity burden. Finally, because actual costs attributable to each type of productivity loss were not available in the database, attributable productivity costs were estimated by multiplying the number of hours/days lost by an estimated hourly wage and therefore did not reflect the true costs to the employer.

## 5 CONCLUSIONS

This real‐world analysis demonstrated that employees with overweight and obesity had higher loss of work productivity as measured by absenteeism, STD, LTD, and WC compared with employees with normal weight. Productivity loss was greater with each higher BMI category both before and after adjusting for differences in age, sex, employer industry, and employment status. In addition, females and older individuals generally had a higher number of hours/days lost and higher attributable productivity costs associated with absenteeism, STD, LTD, and WC. The differences in productivity loss associated with BMI category, sex, and age category may be related to the differences in comorbidity burden among employees with each of these demographic factors.

Overall, the study findings suggest that overweight and obesity, if left untreated, may lead to poorer health and impaired productivity. Therefore, tailored specific interventions should be considered at the workplace to help address this public health crisis and the associated economic burden due to work productivity loss among employees with overweight and obesity. Future studies are warranted to better understand how clinically meaningful weight loss, including intentional weight loss (via anti‐obesity medications, surgery, lifestyle changes, or other methods), may improve health and work productivity in employees with overweight and obesity.

## AUTHOR CONTRIBUTIONS

All authors were involved in the study design, analysis and interpretation of the data, drafting and revising the paper, and approving the final version of the manuscript. All authors agree to be accountable for all aspects of the work.

## CONFLICT OF INTEREST STATEMENT

SS and CJL are employees and stockholders of Eli Lilly and Company. MJ and ATT are employees of Merative, which received funding from Eli Lilly and Company to conduct this study. This research was presented in part at the 2023 Annual Meeting of the Endocrine Society (ENDO 2023) in Chicago, Illinois, US.

## Supporting information

Supporting Information S1

## Data Availability

The data that support the findings of this study are available from Merative via a license which includes terms and conditions around its appropriate use and licensing fees.
