# Supplementary material for: Work loss among privately insured employees with overweight and obesity in the United States
Source: Obes Sci Pract. 2024 Jul 8;10(4):e775. doi: 10.1002/osp4.775 (PMC11229424; doi:10.1002/osp4.775)
Supplement: Supplementary file 1 — Supporting Information S1 [file OSP4-10-e775-s001.docx]

**Title**

Work Loss Among Privately Insured Employees with Overweight and Obesity in the United States

**Authors**

Shraddha Shinde^1^, Michelle Jerry^2^, Anh Thu Tran^2^, Clare J. Lee^1^

^1^Eli Lilly and Company, Indianapolis, USA ​

^2^Merative, Ann Arbor, Michigan, USA​

**Corresponding Author:** Shraddha Shinde

Eli Lilly and Company

Lilly Corporate Center, Indianapolis, IN 46285, USA

[shinde_shraddha@lilly.com](mailto:shinde_shraddha@lilly.com)

# Table S1. Adjusted Mean Ratios of Number of Absence Hours

|  | **Absence Hours**  Mean Ratio (95% CI) | |
| --- | --- | --- |
| BMI Category (Normal Weight) | *Base Category* | |
| BMI Category (Overweight) | 1.03 (1.01-1.04) | |
| BMI Category (Obesity Class 1) | 1.07 (1.05-1.08) | |
| BMI Category (Obesity Class 2) | 1.08 (1.07-1.10) | |
| BMI Category (Obesity Class 3) | 1.14 (1.12-1.15) | |
| Age, in decades | 1.08 (1.08-1.09) | |
| Sex (Male) | *Base Category* | |
| Sex (Female) | 0.99 (0.98-1.00) | |
| Industry (Manufacturing, Durable Goods) | *Base Category* | |
| Industry (Manufacturing, Nondurable Goods) | 0.68 (0.66-0.69) ^1^ | |
| Industry (Transportation, Communications, Utilities) | 0.81 (0.81-0.82) ^1^ | |
| Industry (Finance, Insurance, Real Estate) | N/A^2^ | |
| Industry (Services) | 0.99 (0.98-1.00) ^1^ | |
| Industry (Retail Trade) | N/A^2^ | |
| Industry (Oil & Gas Extraction, Mining) | 0.81 (0.79-0.82) ^1^ | |
| Employment Status (Full-time) | *Base Category* | |
| Employment Status (Part-time) | 0.55 (0.54-0.56) | |
| ^1^The mean ratios for the employer industry variable should be interpreted with caution. Some contributors of MarketScan HPM Database provide all types of absence (e.g., sick, recreational, jury duty, etc.) while others only report some types of absence. Employer industry thus not only reflects differences in absence by industry but also serves as a surrogate for the employer that contributed the data. Although employer industry is an important adjustment variable, the reported ratios should not be interpreted to reflect true industry trends in workplace absence days.  ^2^No employees in the given analysis had this employer industry. | |  |

# Table S2. Adjusted Odds Ratios of Having a Work Loss Claim (Short-term Disability, Long-term Disability, or Workers’ Compensation)

|  | **Any STD Claim**  Odds Ratio (95% CI) | **Any LTD Claim**  Odds Ratio (95% CI) | **Any WC Claim**  Odds Ratio (95% CI) | |
| --- | --- | --- | --- | --- |
| BMI Category (Normal Weight) | *Base Category* | | | |
| BMI Category (Overweight) | 1.13 (1.10-1.17) | 0.93 (0.82-1.06) | 1.04 (0.98-1.10) | |
| BMI Category (Obesity Class 1) | 1.45 (1.41-1.50) | 1.05 (0.92-1.20) | 1.26 (1.19-1.34) | |
| BMI Category (Obesity Class 2) | 1.80 (1.74-1.86) | 1.27 (1.10-1.46) | 1.36 (1.28-1.45) | |
| BMI Category (Obesity Class 3) | 2.88 (2.79-2.98) | 1.93 (1.68-2.21) | 1.36 (1.27-1.45) | |
| Age, in decades | 1.16 (1.15-1.17) | 1.50 (1.44-1.57) | 1.04 (1.02-1.06) | |
| Sex (Male) | *Base Category* | | | |
| Sex (Female) | 1.37 (1.34-1.40) | 1.25 (1.15-1.36) | 1.18 (1.13-1.23) | |
| Industry (Manufacturing, Durable Goods) | *Base Category* | | | |
| Industry (Manufacturing, Nondurable Goods) | 0.55 (0.53-0.57) | 0.56 (0.48-0.64) | 0.64 (0.59-0.69) | |
| Industry (Transportation, Communications, Utilities) | 0.70 (0.69-0.72) | 0.95 (0.87-1.04) | 1.21 (1.16-1.26) | |
| Industry (Finance, Insurance, Real Estate) | 0.53 (0.51-0.54) | 0.57 (0.49-0.65) | 0.06 (0.05-0.08) | |
| Industry (Services) | 0.39 (0.38-0.41) | 0.36 (0.32-0.42) | 0.86 (0.82-0.91) | |
| Industry (Retail Trade) | 0.28 (0.27-0.30) | 0.14 (0.10-0.21) | N/A^1^ | |
| Industry (Other/Unknown^2^) | 0.08 (0.07-0.10) | 0.07 (0.03-0.15) | 0.07 (0.05-0.09) | |
| Employment Status (Full-time) | *Base Category* | | | |
| Employment Status (Part-time) | 0.77 (0.69-0.86) | 0.30 (0.18-0.47) | 1.69 (1.55-1.85) | |
| ^1^No employees in the given analysis had this employer industry.  ^2^Depending on the analysis, this category may include any combination of agriculture/forestry/fishing, construction, oil & gas extraction/mining, wholesale, and unknown. | | | |  |

# Figure S1. Baseline Clinical Characteristics Stratified by Sex

1A. CCI score

1B. Number of comorbidities

# Figure S2. Baseline Clinical Characteristics Stratified by Age Category

2A. CCI score

2B. Number of comorbidities
